# Supplementary material for: Trajectory patterns of SARS-CoV-2 neutralising antibody response in convalescent COVID-19 patients
Source: Commun Med (Lond). 2022 May 19;2:53. doi: 10.1038/s43856-022-00119-2 (PMC9120513; doi:10.1038/s43856-022-00119-2)

## Supplementary Information

# Trajectory patterns of SARS-CoV-2 neutralising antibody response in convalescent COVID-19 patients

**Ngai Sze Wong,[1,2&] Shui Shan Lee,[1&] Denise P.C. Chan,[1] Timothy C.M. Li,[3] Tracy H.Y. Ho,[3] Fion W.L. Luk,[3] Kai Ming Chow,[3] Eugene Y.K. Tso,[4] Eng-Kiong Yeoh,[2,5] Samuel Y.S. Wong,[2] David S.C. Hui,[3] Grace C.Y. Lui[3]***

[&] These authors contributed equally.

*** Corresponding Author: Grace C.Y. Lui**
Department of Medicine and Therapeutics, The Chinese University of Hong Kong, 9/F Lui Che Woo Clinical Sciences Building, Prince of Wales Hospital, Shatin, Hong Kong, China; E-mail: gracelui@cuhk.edu.hk

[1] Stanley Ho Centre for Emerging Infectious Diseases, The Chinese University of Hong Kong, Shatin, Hong Kong, China
[2] The JC School of Public Health and Primary Care, The Chinese University of Hong Kong, Shatin, Hong Kong, China
[3] Department of Medicine and Therapeutics, The Chinese University of Hong Kong, Shatin, Hong Kong, China
[4] Department of Medicine & Geriatrics, United Christian Hospital, Hong Kong, China
[5] Centre for Health Systems and Policy Research, The Chinese University of Hong Kong, Shatin, Hong Kong, China

**Supplementary Figure 1. sVNT (%) trajectory of each patient by identified two classes in latent class mixed models**

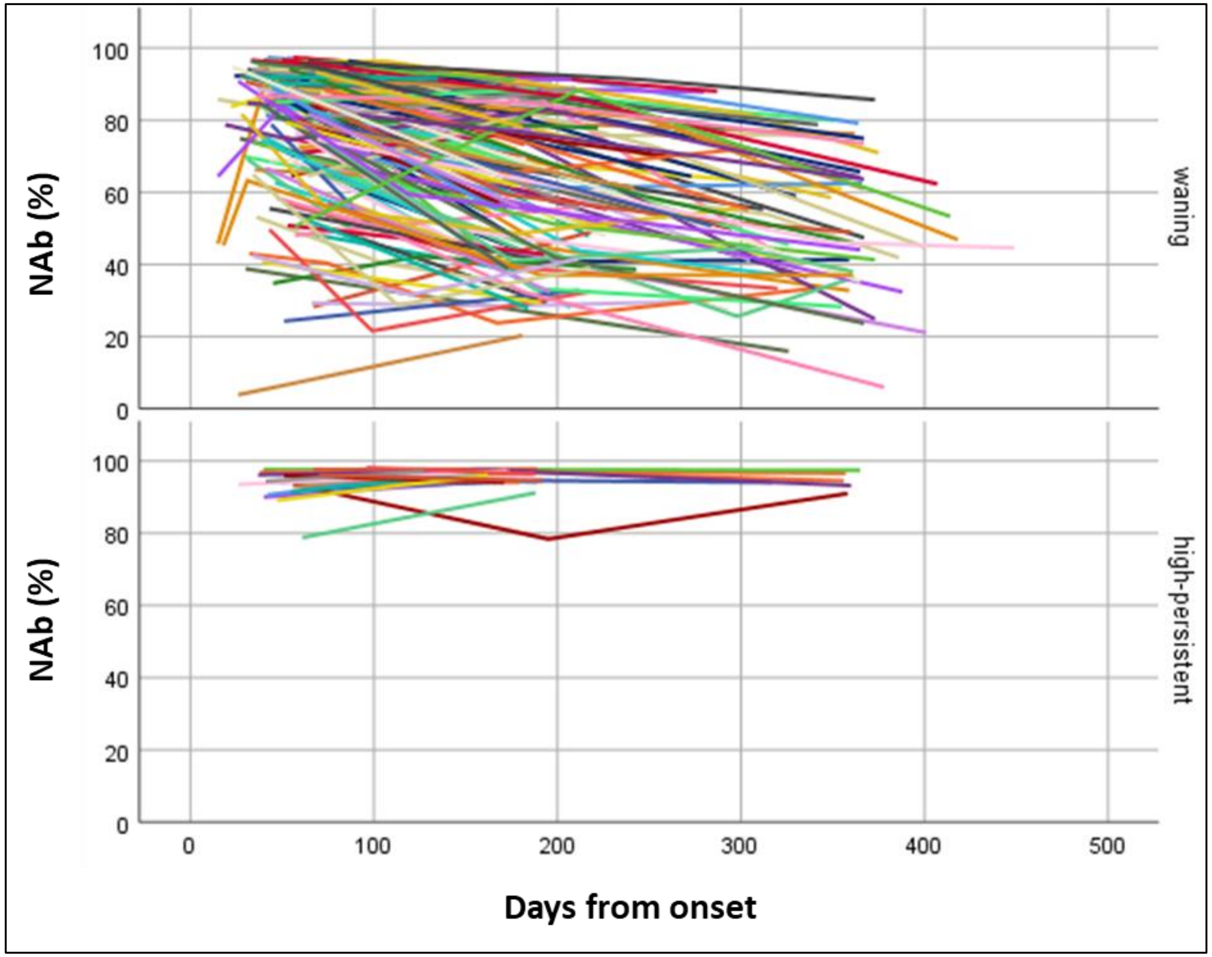

**Supplementary Figure 2. Scattered plot for IgG spike ratio over days from symptom onset by the two classes identified in latent class mixed models**

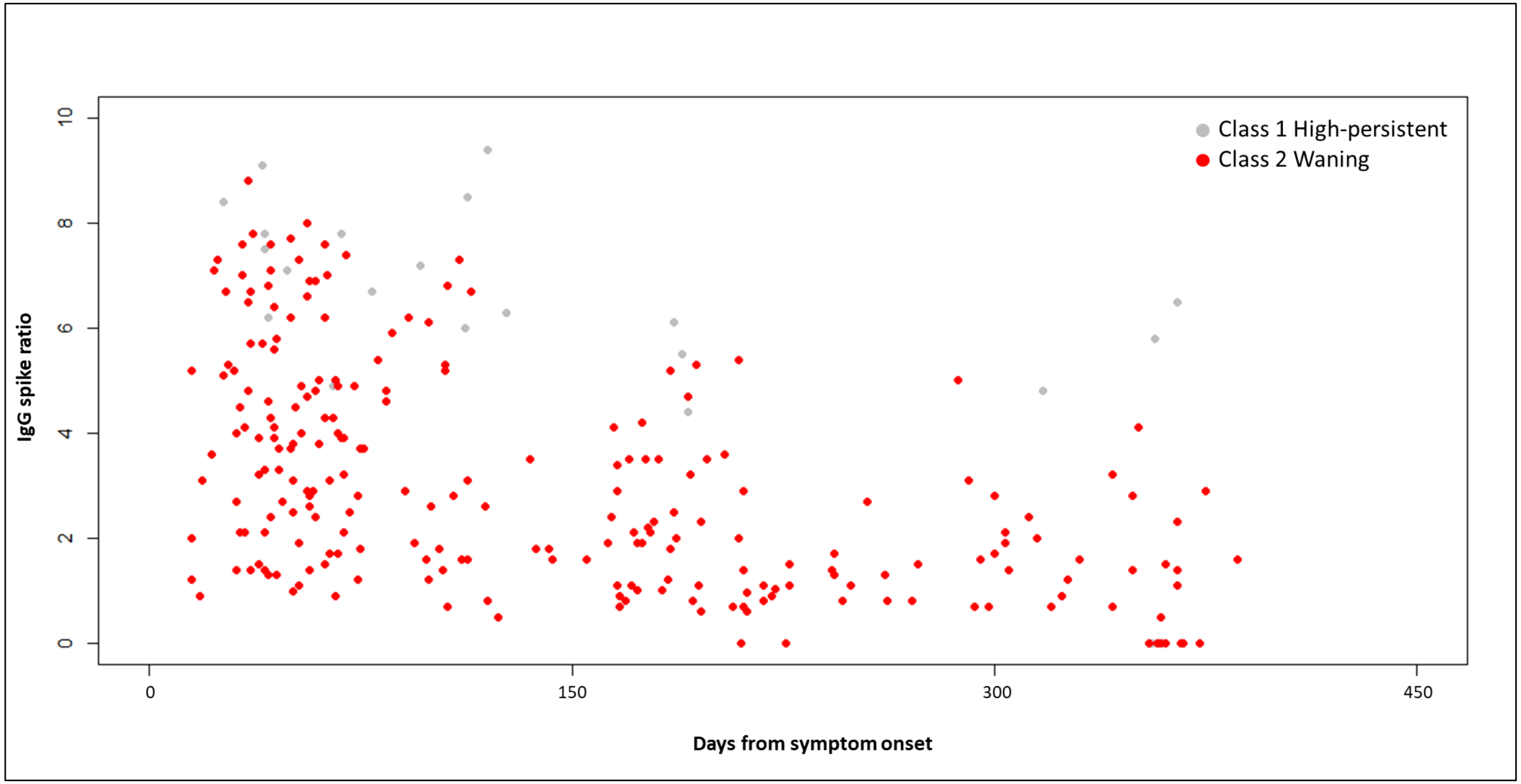


IgG Spike TEST is semiquantitative, the calculation of a ratio from the test and the calibrator

**Supplementary Figure 3. The median sVNT (%) trajectory line chart with 95% CI presented as error bar by identified classes in sensitivity analyses**

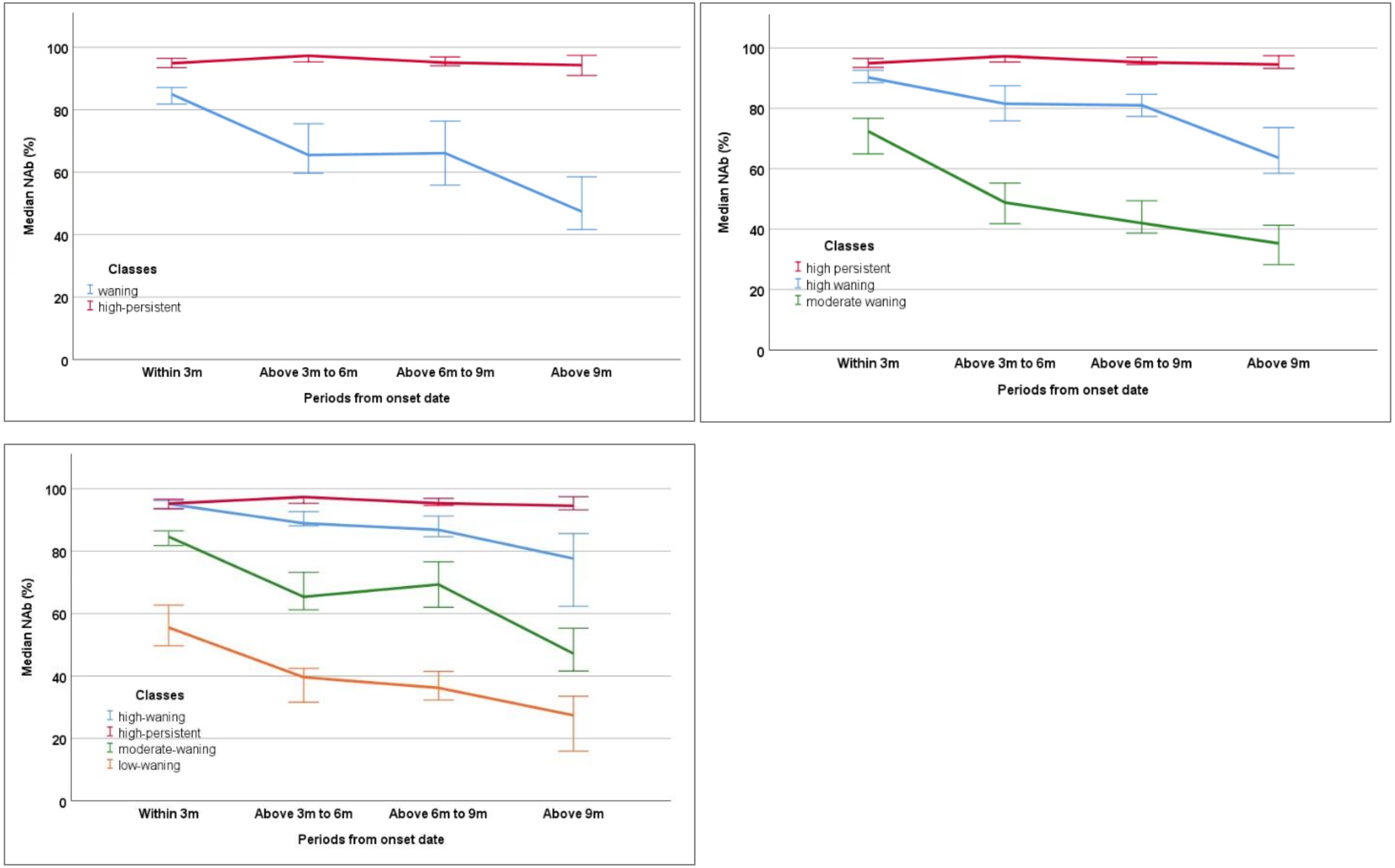

Supplement: Supplementary file 1 — Supplementary information [file 43856_2022_119_MOESM1_ESM.pdf]
