# Supplementary material for: Trajectory patterns of SARS-CoV-2 neutralising antibody response in convalescent COVID-19 patients
Source: Commun Med (Lond). 2022 May 19;2:53. doi: 10.1038/s43856-022-00119-2 (PMC9120513; doi:10.1038/s43856-022-00119-2)
Supplement: Supplementary file 2 — Description of Additional Supplementary Files [file 43856_2022_119_MOESM2_ESM.pdf]

Supplementary Data

Supplementary Data 1. General characteristics of SARS-CoV-2 patients (n=368)

Supplementary Data 2. Comparison between patients aged below 60 (n=265) and aged 60 or above (n=103)

Supplementary Data 3. Proportion of patients positive for serology markers of IgG NP, IgG spike, and neutralising antibody (NAb) with surrogate virus neutralisation test (sVNT)

Supplementary Data 4. Latent class mixed models model summaries by number of classes and non-linear functions

Supplementary Data 5. Comparison of characteristics by two classes identified in latent class mixed model (LCMM), n=200

Supplementary Data 6. Comparison of characteristics by three classes identified in latent class mixed model (LCMM), a sensitivity analysis

Supplementary Data 7. Comparison of characteristics by class in 4-class latent class mixed model (LCMM), n=200

Supplementary Data 8. Factors associated with class identity in multivariable multinominal logistic regression

Supplementary Data 9. Source data for Figures 1-3
