# Supplementary material for: Trajectory patterns of SARS-CoV-2 neutralising antibody response in convalescent COVID-19 patients
Source: Commun Med (Lond). 2022 May 19;2:53. doi: 10.1038/s43856-022-00119-2 (PMC9120513; doi:10.1038/s43856-022-00119-2)
Supplement: Supplementary file 13 — Reporting Summary [file 43856_2022_119_MOESM13_ESM.pdf]

nature research

Corresponding author(s): GL

Last updated by author(s): Apr 23, 2022

Reporting Summary

Nature Research wishes to improve the reproducibility of the work that we publish. This form provides structure for consistency and transparency in reporting. For further information on Nature Research policies, see our Editorial Policies and the Editorial Policy Checklist.

Statistics

For all statistical analyses, confirm that the following items are present in the figure legend, table legend, main text, or Methods section.

n/a	Confirmed	
☐	☒	The exact sample size (*n*) for each experimental group/condition, given as a discrete number and unit of measurement
☐	☒	A statement on whether measurements were taken from distinct samples or whether the same sample was measured repeatedly
☐	☒	The statistical test(s) used AND whether they are one- or two-sided<br>*Only common tests should be described solely by name; describe more complex techniques in the Methods section.*
☐	☒	A description of all covariates tested
☒	☐	A description of any assumptions or corrections, such as tests of normality and adjustment for multiple comparisons
☐	☒	A full description of the statistical parameters including central tendency (e.g. means) or other basic estimates (e.g. regression coefficient) AND variation (e.g. standard deviation) or associated estimates of uncertainty (e.g. confidence intervals)
☒	☐	For null hypothesis testing, the test statistic (e.g. *F*, *t*, *r*) with confidence intervals, effect sizes, degrees of freedom and *P* value noted<br>*Give P values as exact values whenever suitable.*
☐	☒	For Bayesian analysis, information on the choice of priors and Markov chain Monte Carlo settings
☐	☒	For hierarchical and complex designs, identification of the appropriate level for tests and full reporting of outcomes
☒	☐	Estimates of effect sizes (e.g. Cohen's *d*, Pearson's *r*), indicating how they were calculated

Our web collection on statistics for biologists contains articles on many of the points above.

Software and code

Policy information about availability of computer code

Data collection	Excel
Data analysis	R: lcmm package; ggplot2 package<br>SPSS 25:

For manuscripts utilizing custom algorithms or software that are central to the research but not yet described in published literature, software must be made available to editors and reviewers. We strongly encourage code deposition in a community repository (e.g. GitHub). See the Nature Research guidelines for submitting code & software for further information.

Data

Policy information about availability of data

All manuscripts must include a data availability statement. This statement should provide the following information, where applicable:

- Accession codes, unique identifiers, or web links for publicly available datasets
- A list of figures that have associated raw data
- A description of any restrictions on data availability

The dataset cannot be deposited in a public repository because sharing of primary data is restricted by the Joint Chinese University of Hong Kong – New Territories East Cluster Clinical Research Ethics Committee due to confidentiality concerns on patient data. Anonymous clinical and laboratory data are owned by the Hospital Authority, while anonymous surveillance data are owned by the Department of Health, Hong Kong SAR Government, to which inquiry for access and permission for research purpose could be directed. However, the source data for the main figures are available as Supplementary Data 9.

Field-specific reporting

Please select the one below that is the best fit for your research. If you are not sure, read the appropriate sections before making your selection.

☐ Life sciences ☒ Behavioural & social sciences ☐ Ecological, evolutionary & environmental sciences

For a reference copy of the document with all sections, see nature.com/documents/nr-reporting-summary-flat.pdf

Behavioural & social sciences study design

All studies must disclose on these points even when the disclosure is negative.

Study description	A quantitative longitudinal study
Research sample	subject: COVID-19 patients in Hong Kong<br>485 patients were recruited, and 368 with IgG NP and NAb measurements in day 15-90 were selected for analysis:<br>51% were male, 28% aged 60 or above (median 48 years old, IQR 31-61)<br>Patients were recruited from the Prince of Wales Hospital, a tertiary hospital in the public service with catchment for some 1.8 million population in the New Territories East Region of Hong Kong.
Sampling strategy	All consecutive patients admitted to the study site and were eligible were recruited into this study. The sample size was similar or greater than most other studies evaluating longitudinal immunological response in COVID-19 patients.
Data collection	Blood sampling was performed at multiple time points during hospitalisation and follow-ups after discharge. Written informed consent was obtained from each participant. The sociodemographic variables including gender, age, onset date, reporting date, hospital admission and discharge dates, travel history (e.g. origin and mode of travel), and epidemiological linkage data were collected by research staff through face-to-face interview, following a structured questionnaire. Data were input in Excel.
Timing	Between February 2020 and February 2021
Data exclusions	Patients without both IgG NP and NAb measured between day 15 and 90 from onset date for symptomatic patients or reporting date for asymptomatic patients were excluded in analysis
Non-participation	no participants dropped out / declined participation
Randomization	participants were not allocated into experimental groups

Reporting for specific materials, systems and methods

We require information from authors about some types of materials, experimental systems and methods used in many studies. Here, indicate whether each material, system or method listed is relevant to your study. If you are not sure if a list item applies to your research, read the appropriate section before selecting a response.

Materials & experimental systems

n/a	Involved in the study	
☒	☐	Antibodies
☒	☐	Eukaryotic cell lines
☒	☐	Palaeontology and archaeology
☒	☐	Animals and other organisms
☐	☒	Human research participants
☐	☒	Clinical data
☒	☐	Dual use research of concern

Methods

n/a	Involved in the study	
☒	☐	ChIP-seq
☒	☐	Flow cytometry
☒	☐	MRI-based neuroimaging

Human research participants

Policy information about studies involving human research participants

Population characteristics	Adult patients hospitalized with COVID-19
Recruitment	Consecutive adult patients admitted to study site were invited to participate in this study. All eligible patients who consented were recruited into this study.
Ethics oversight	the Joint Chinese University of Hong Kong – New Territories East Cluster Clinical Research Ethics Committee (CREC)

Note that full information on the approval of the study protocol must also be provided in the manuscript.

Clinical data

Policy information about clinical studies

All manuscripts should comply with the ICMJE guidelines for publication of clinical research and a completed CONSORT checklist must be included with all submissions.

Clinical trial registration	NCT05028881
Study protocol	Study protocol is not available as this is not a clinical trial. No intervention is involved.
Data collection	Patients were recruited from the Prince of Wales Hospital, a tertiary hospital in the public service in Hong Kong, between February 2020 and February 2021.
Outcomes	The main outcome was neutralising antibody level, and the secondary outcomes were IgG spike and IgG NP detection
